# The impact of the use of bioresorbable vascular scaffolds and drug-coated balloons in coronary bifurcation lesions

**DOI:** 10.1186/s43044-019-0033-z

**Published:** 2019-12-16

**Authors:** Mostafa Elwany, Amr Zaki, Azeem Latib, Luca Testa, Alfonso Ielasi, Davide Piraino, Salvatore Geraci, Tarek El Zawawy, Bernardo Cortese

**Affiliations:** 1 Interventional Cardiology, San Carlo Clinic, Milano, Italy; 2 Faculty of Medicine, University of Alexandria, Alexandria, Egypt; 3 Montefiori Medical Center, New York, USA; 4 San Donato Clinic, Milano, Italy; 5 Sant’Ambrogio Clinic, Milano, Italy; 6 Policlinico Giaccone, Palermo, Italy; 7 Ospedale di Agrigento, Agrigento, Italy; 8 Fondazione G. Monasterio CNR-Regione Toscana, Pisa, Massa, Italy

**Keywords:** Bioresorbable vascular scaffolds (BVS), Drug-coated balloons (DCB), Coronary bifurcation lesions

## Abstract

**Background:**

Despite the improvement in techniques and tools, coronary lesions involving a bifurcation are still challenging and the outcome with drug-eluting stents is not always optimal. The role of bioresorbable vascular scaffolds (BVS) and drug-coated balloons (DCB) in this setting has not been adequately investigated yet.

**Results:**

From the databases of 6 italian centers with high proficiencies in newer technologies, we retrospectively collected all consecutive cases of coronary bifurcations managed or attempted with the implantation of at least one BVS in the main vessel and the use of one DCB in the side branch (SB). Primary study endpoint was the occurrence of major adverse cardiovascular events (MACE) at the longest available follow-up. Fourty patients fulfilled the enrollment criterion, 22.5% had diabetes and 50% an acute coronary syndrome. Average syntax score was 15.04 ± 7.18, all lesions were de novo, and 27 patients (67.5%) had a type 1,1,1 Medina lesion. Twenty-three lesions (57.5%) involved the proximal left anterior-descending artery/first diagonal branch. Only 32.5% of patients underwent an intravascular imaging-guided angioplasty. Average lesion length was 21.4 mm in the main vessel and 11.49 mm in the SB. MV was always predilated and BVS received a postdilation in 100% of the cases. In 42.5% of the cases, the DCB was used during final kissing balloon inflation, and in no cases, a stent/BVS was required in the SB. Procedural success was achieved in 100% of the cases. After an average follow-up of 15.5 (± 11.5) months, we observed no MACE with only one case of target vessel revasularization (2.5%).

**Conclusions:**

Management of coronary bifurcation lesions with the use of newer technologies including BVS and DCB seems feasible and effective at mid-term and long-term clinical follow-up.

## Background

Despite the advances in the field of interventional cardiology, coronary bifurcations lesions, which represent approximately 15–25% of percutaneous coronary interventions (PCI) cases, are still a challenge [4].

Bioresorbable vascular scaffolds (BVS), which dissolve after fulfilling their support function have been a perennial aim and their introduction to the field of interventional cardiology represented a revolution and hope for vascular reparative therapy [8].

The Absorb BVS (Abbott Vascular, Santa Clara, CA, USA) is a fully bioresorbable scaffold where the resorption process progresses gradually, mainly secondary to hydrolysis creating minimal or no inflammation. One of the major limitations of the BVS is the 157 microns strut thickness [14] which make it bulky .

It is reasonable to expect that the theoretical advantages of BVS over metallic drug eluting stents (DES) are to be more pronounced in the subset of coronary bifurcation lesions. Several reasons make us believe in this conclusion; first, arterial healing is faster with the BVS than DES especially if a 2 stent technique was used. Second, late luminal enlargement is secondary to BVS degradation. Third, jailing of the SB is no longer permanent thanks to the resorption of the BVS [15].

Provisional approach remains the gold standard for percutaneous treatment of patients with unselected bifurcated lesions even when the use of BVS is intended [1].

Side branch (SB) management is still a challenge. The use drug-coated balloon (DCB) for addressing such an issue may prove advantageous as compared to regular balloon angioplasty [10].

Our aim in this study was to evaluate the performance of BVS and DCB in bifurcation lesions at midterm follow-up in order to gain a better understanding of their efficacy and safety at this clinical setting.

## Methods

The study is a retrospective study where patients were enrolled from 6 Italian centers over the period from July 2013 to July 2017 with at least 6 months follow-up after the index procedure of the last patient to the maximum available follow-up. The study included all consecutive cases of coronary bifurcations managed or attempted with:

(a) The implantation of at least one Absorb BVS in the main vessel.

(b) The use of one or more DCB in the side branch.

### Exclusion criteria

1. Cardiogenic shock
2. Severe renal impairment (creatinine clearance < 30 ml/min) or dependence on dialysis.
3. Contraindication to prolonged dual antiplatelet therapy.

Clinical data, including age, sex, risk factors (hypertension, diabetes, dyslipidemia, smoking and family history) and history (previous MI, previous PCI, previous bypass surgery, cerebrovascular disease CABG) was thoroughly obtained. The clinical indication (chronic stable angina, unstable angina, STEMI, or NSTEMI) was also included in the study. Renal function was withdrawn and transthoracic echocardiography was performed in all patients.

The PCI procedural details were also recorded including type of bifurcation lesion (according to Medina classification) [12], Syntax score, intravascular imaging, balloon predilation, BVS, and DCB used and balloon postdilatation.

Follow-up at the maximum available timing with a minimum of 6 months was done for major adverse cardiovascular events including death, non-fatal MI, scaffold thrombosis, and cerebrovascular stroke.

## Results

**Fig. 1:**
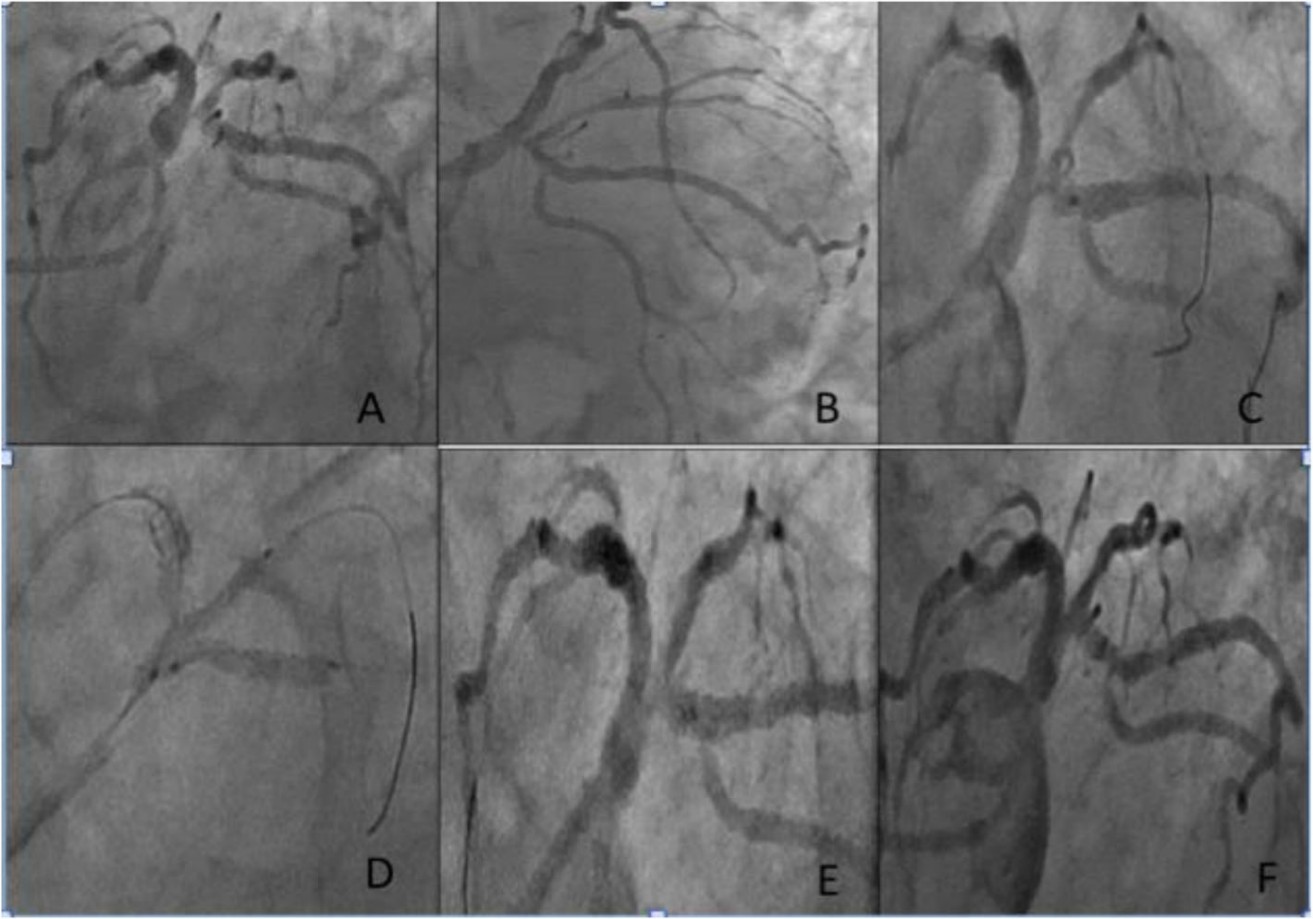
**a**, **b** Bifurcation lesion involving the ostia of the LCX and RI. **c** After deployment of the BVS. **d** Kissing balloon inflation with DCB. **e** Final result at the index procedure. **f** At follow-up after 25 months

Forty patients fulfilled the enrollment criteria, 22.5% had diabetes and 50% an acute coronary syndrome at presentation. The demographic and clinical characteristics of the patients enrolled in the study are enlisted in Table 1. Average syntax score was 15.04 ± 7.18, all lesions were de novo, and 27 patients (67.5%) had a type 1,1,1 Medina lesion. Twenty-three lesions (57.5%) involved the proximal left anterior-descending artery/first diagonal branch. Only 32.5% of patients underwent an intravascular imaging-guided angioplasty. Average lesion length was 21.4 mm in the main vessel and 11.5 mm in the SB. MV was always predilated and BVS received a postdilation in 100% of the cases. In 42.5% of the cases, the DCB was used during final kissing balloon inflation, and in no cases, a stent/BVS was required in the SB. Procedural success was achieved in 100% of the cases. The procedural characteristics are enlisted in Table 2. After an average follow-up of 15.5 (± 11.5) months, we observed no MACE with only one case of target vessel revascularization (2.5%) as shown in Table 3. All the DCBs used eluted paclitaxel (Fig. 1).

**Table 1:** Demographic and clinical characteristics of the study group

Criterion	BVS & DCB group (*n* = 40)
Males	34 (85%)
Mean age	56.9 ± 10.3
Hypertension	23 (57.5%)
DM	9 (22.5%)
IDDM	5(12.5%)
Smoking	14 (35%)
Dyslipidemia	22 (55%)
CABG	1 (2.5%)
Prior PCI	11 (27.5%)
Stroke	0%
Previous MI	7 (17.5%)
Creatinine	0.92 ± 0.16 mg/dl
HB	13.3 ± 1.13 md/dl
Weight	79 ± 12 kg
Height	170.9 ± 6.1 cm
Ejection fraction	56.6 ± 5.1
Clinical indication	
Stable angina	20 (50%)
Unstable angina	6 (15%)
NSTEMI	8 (20%)
STEMI	6 (15%)

**Table 2:** Procedural details

Procedural details	BVS & DCB group (*n* = 40)
Access site	
Radial	32 (80%)
Femoral	8 (20%)
LM diseased	1 (2.5%)
LAD diseased	23 (57.5%)
LCX diseased	11 (27.5%)
RCA diseased	8 (20%)
Syntax score	15.04 ± 7.18
Medina class	
1,1,1	27(67.5%)
1,1,0	0(0%)
1,0,1	0%
0,1,1	9 (22.5%)
0,1,0	1 (2.5%)
1,0,0	1 (2.5%)
0,1,1	2 (5%)
OCT	3 (7.5%)
IVUS	10 (25%)
ACC/AHA	
A	0%
B1	2 (5%)
B2	34 (85%)
C	4 (10%)
Denovo lesions	100%
ISR	0%
Thrombus	5 (2.5%)
CTO	6 (15%)
RVD (proximal MV) (mm)	3.13 ± 0.4
Lesion length (MV) (mm)	21.42 ± 16.25
RVD SB (mm)	2.31 ± 0.34
MLD MV (mm)	0.51 ± 0.37
MLD SB (mm)	0.77 ± 0.62
Lesion length (SB) (mm)	11.49 ± 6.35
% stenosis MV	83.2 ± 13.4%
% stenosis SB	66.79 ± 24%
Predilatation MV	40 (100%)
Predilatation MV balloon diameter	2.81 ± 0.45 m
Predilatation pressure (atm)	13 ± 0.36
Scoring balloon MV	1 (2.5%)
Rotablator MV	0%
Type of BVS	Absorb (100%)
Length BVS (mm)	22.02 ± 6.07
Second BVS used	7 (17.5%)
Inflation pressure BVS (atm)	10.92 ± 1.4 atm
Post dilatation of BVS	40 (100%)
Post dilatation balloon diameter (mm)	3.31 ± 0.39
Inflation pressure of postdilatation balloon (atm)	20.3 ± 4.4
Predilatation SB	32 (80%)
KB before stent implantation	5 (12.5%)
Kissing balloon inflation	23(57.5%)
Predilatation of SB balloon diameter	2.25 ± 0.33
Predilatation SB balloon inflation pressure	11 ± 3 atm
Type of DCB used	
Pantera Lux	6 (15%)
Elutax SV	11 (27.5%)
Restore	6 (15%)
Sequent please	4 (10%)
In.Pact Falcon	10 (25%)
Danubu	1 (2.5%)
Agent	1(2.5%)
Magic	1 (2.5%)
Diameter of DCB	2.43 ± 0.37 mm
DCB length	20.36 ± 6.42 mm
DCB inflation pressure	9.4 ± 1.9 atm
DCB inflation duration	52 ± 10 sec
FKB with normal balloons	6 (15%)
FKB with DCB	17 (42.5%)
Dissection left after DCB	6 (15%)
Type of dissections	
A	5 (12.5%)
C	1 (2.5%)
Stenting of SB	0%
Final % diameter stenosis MB	5.3 ± 8%
Final % diameter stenosis SB	13.0 ± 16.4%
Final MLD MB (mm)	3.0 ± 0.43 mm
Final MLD SB (mm)	1.9 ± 0.5 mm
Total amount of contrast (ml)	212 ± 117
Procedural time (min)	71.8 ± 38 min
Total fluoroscopy time (min)	12 ± 3.9 min
BVS/DES-related complications	0%
BVS underexpansion	0%
Longitudinal deformation	0%
BVS recoil	0%
Final TIMI less than 3	0%
Intraprocedural occlusiom	0%
Intraprocedural death	0%
Peri-procedural MI	0%
Medications at discharge	
Aspirin	40(100%)
Clopidogrel	11(27.5%)
Prasugrel	4 (10%)
Ticagrelor	25(62.5%)

**Table 3:** Follow-up

Follow-up	*n* = 40
Angiographic follow-up	13 (32.5%)
Angiographic follow-up indication	
Stable angina	8 (9%)
Unstable angina	3 (7.5%)
STEMI	1 (2.5%)
NSTEMI	1 (2.5%)
% diameter stenosis of MV in case of angio follow-up	2.9 ± 3.4%
% diameter stenosis of SB	11 ± 29.49%
Average duration from index procedure to the last follow-up (days)	444 ± 303
Binary restenosis MV	0%
Binary restenosis SB	1 (2.5%)
MV MLD	3.2 ± 0.47 mm
SB MLD	1.784 ± 0.6 mm
MV TLR	0%
SB TLR	0%
Aspirin at follow-up	40 (100%)
P2Y12 inhibitors at follow-up	25 (62.5%)
Death	0%
CV death	0%
Non CV death	0%
TV MI	1 (2.5%)
TV MI management	POBA
TLR	0%
TVR	1(2.5%)
Date of TVR	10-4-2014
TLR or TVR management	POBA
TL thrombosis	0%

## Discussion

The BVS was expected to represent fourth revolution in interventional cardiology as it offers a new technology by transient scaffolding the vessel and eluting an antiproliferative drug [13].

Cohort B study, which tested the second generation of BVS, showed a MACE rate of 9.0% [17]. The follow-up after 3 years in ABSORB II revealed a higher rate of target lesion failure (TLF) in the BVS group (7%) [6].

A safety alert was issued after Food and Drug Administration (FDA) reviewed the 2-year data from the ABSORB III trial showing a rate of 11% in major cardiac events in patients treated with BVS in comparison to a rate of 7.9% in patients treated with Everolimus eluting stent (EES) [16].

Thrombosis was the key limitation of the BVS. This was very evident in the Absorb II trial. Specifically, 6 events occurred beyond the first year. The analysis of the 6 cases showed that the main reasons of such events were very late scaffold thrombosis and undersized scaffolds [3].

Amsterdam Investigator-Initiated Absorb Strategy All-Comers Trial (AIDA) found that there was no significant difference in the rate of target-vessel failure (TVF) between the BVS group and the stent group. There was a higher incidence of device thrombosis with BVS throughout the 2-year follow-up period [20].

Optimal management for BVS failure is still a topic for research. Different coronary devices were used to address this issue. Restenosis was successfully managed by percutaneous balloon angioplasty (POBA) and DCB [11] [7, 9].

The FDA recommended the *PSP* technique for BVS implantation. This technique includes 3 steps: the first step is the lesion preparation with a 1:1 balloon-to-artery ratio using a non-compliant balloon. The second step is the appropriate sizing of the vessel with liberal use of intravascular imaging or quantitative coronary angiography (QCA). The third step is the postdilatation to high pressure using a non-compliant balloon up to 0.5 mm above nominal scaffold diameter. The operators were also advised to use the BVS in vessels with a reference diameter of ≥ 2.5 mm and ≤ 3.75 [18].

There are a number of advantages in DCBs make them of great use in SB management in the setting of bifurcation PCI. First, the homogeneous administration of the drug; second, high concentrations of drug are delivered into the vessel wall at the moment of injury; third, original anatomy of the carina is respected [2].

Early experiences have shown how leaving a dissection after plain old balloon angioplasty was associated with increased rates of thrombotic events, early reocclusion, and recurrence of restenosis, and this was one of the main indications for the use of stents in an earlier era. Paclitaxel, when correctly delivered to the vessel wall, may have a role in facilitating the healing of coronary vessels. If the dissection is of low-medium grade, it seems safe to leave it untreated. In fact, data from the literature show how any stent strategy associated with DCB use is unsafe or yields unsatisfactory results. In a consecutive series of patients treated with new-generation DCB for native coronary artery disease and with a final non-flow-limiting dissection, these lesions tended to heal despite their initial severity. After DCB angioplasty, a strategy of bailout stenting should be reserved to more severe, flow-limiting dissections, and in our study, all the dissections were non-flow-limiting, so no DES were needed [5].

### In the time-varying outcomes with the absorb bioresorbable vascular scaffold during a 5-year follow-up: a systematic meta-analysis and individual patient data pooled study

Target lesion failure occurred in 11.6% of BVS-treated patients vs 7.9% of EES-treated patients between 0 and 3 years (HR, 1.42; 95% CI, 1.12–1.80), and 4.3% of BVS-treated patients vs 4.5% of EES-treated patients between 3 and 5 years (HR, 0.92; 95% CI, 0.64–1.31) (*P* for interaction = .046). Device thrombosis occurred in 2.4% of BVS-treated patients vs 0.6% of EES-treated patients between 0 and 3 years (HR, 3.86; 95% CI, 1.75–8.50) and 0.1% of BVS-treated patients vs 0.3% of EES-treated patients between 3 and 5 years (HR, 0.44; 95% CI, 0.07–2.70) (*P* for interaction = .03). This study shows that despite the worse performance of the BVS as regards TLR and scaffold thrombosis over 0–3 years, their performance was non-inferior to DES or even better as regards TLR and thrombosis over 3–5 years. This gives a hope for the return of the BVS to routine clinical practice after overcoming the technical and procedural issues that influence its safety and efficacy [19].

The idea of “leaving nothing behind” after PCI is a very exciting concept in modern interventional cardiology especially in bifurcation lesions. This dream started to come true with the introduction of BVS and DCB to the field of interventional cardiology which are still understudied and they open the door for further research in these technologies.

## Conclusion

Our knowledge about the BVS and DCB technology is still growing. However, as it occurred with the first generation of DES, we are still learning how to appropriately use the BVS.

Management of coronary bifurcation lesions with the use of newer technologies including BVS and DCB was found to be feasible and effective at mid-term and long-term clinical follow-up with the implementation of proper implantation techniques.

## Data Availability

The datasets used and/or analyzed during the current study are available from the corresponding author on reasonable request.

## Abbreviations

BVS: Bioresorbable vascular scaffolds
CABG: Coronary artery bypass graft
CAD: Coronary artery disease
CTO: Chronic total occlusion
DCB: Drug-coated balloons
DES: Drug eluting stents
EES: Everolimus eluting stents
ISR: In-stent restenosis
KB: Kissing balloons
MACE: Major adverse cardiovascular events
MB: Main branch
MI: Myocardial infarction
MI: Myocardial infarction
MLD: Minimal lumen diameter
NSTEMI: Non-ST segment elevation myocardial infarction
PCI: Percutaneous coronary intervention
POBA: Percutaneous balloon angioplasty
QCA: Quantitative coronary angiography
RVD: Residual vessel diameter
SB: Side branch
STEMI: ST segment elevation myocardial infarction
TIMI: Thrombolysis in myocardial infarction
TLR: Target lesion revascularization
TVR: Target vessel revascularization
